# Supplementary figures and images for: Novel viral splicing events and open reading frames revealed by long-read direct RNA sequencing of adenovirus transcripts
Source: PLoS Pathog. 2022 Sep 12;18(9):e1010797. doi: 10.1371/journal.ppat.1010797 (PMC9499273; doi:10.1371/journal.ppat.1010797)

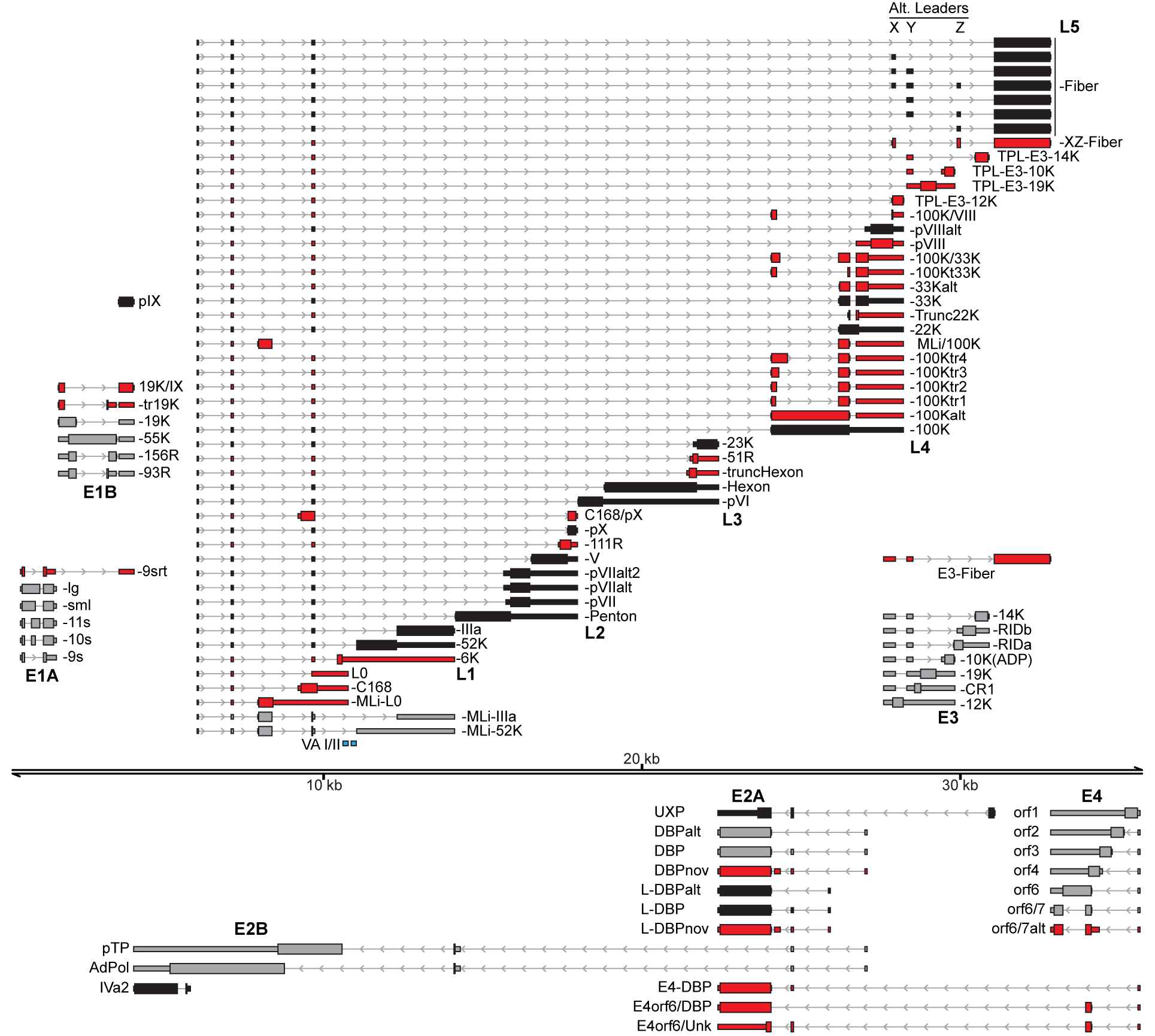

Supplement: S1 Fig — Expanded transcriptome showing all high confidence transcripts. Color scheme is the same as Fig 2. All transcripts are labeled as denoted in Fig 3. (TIF) [file ppat.1010797.s003.tif]

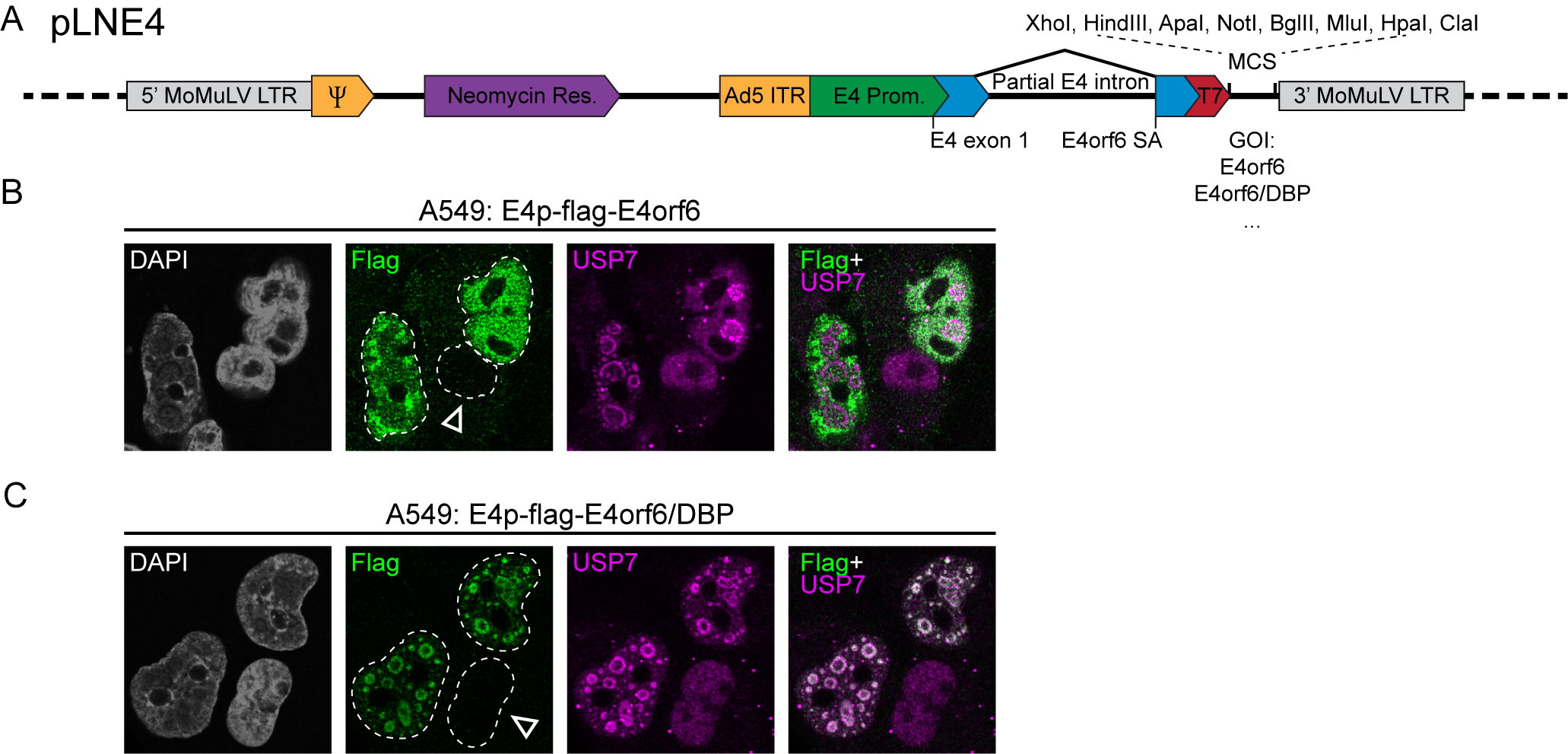

Supplement: S2 Fig — (A) The retroviral plasmid pLNE4 was designed to allow the expression of transgenes under the control of the Ad5 E4 promoter. This has the effect of only expressing transgenes after viral infection and E1A expression, thus allowing for the regulated expression of potentially toxic viral proteins. (B) A549 cells were transduced with a pLNE4 vector expressing flag-tagged E4orf6. Uninfected cells (open arrowhead) show no expression of flag-tagged proteins. DAPI staining marks nuclei. (C) Same as in panel B, but A549 cells were transduced with a pLNE4 vector expressing flag-tagged E4orf6/DBP. While E4orf6 is broadly nucleoplasmic, E4orf6/DBP strongly localizes to viral replication centers marked by the cellular protein USP7 (magenta). Immunofluorescence was performed 24 hours post-infection. (TIF) [file ppat.1010797.s004.tif]

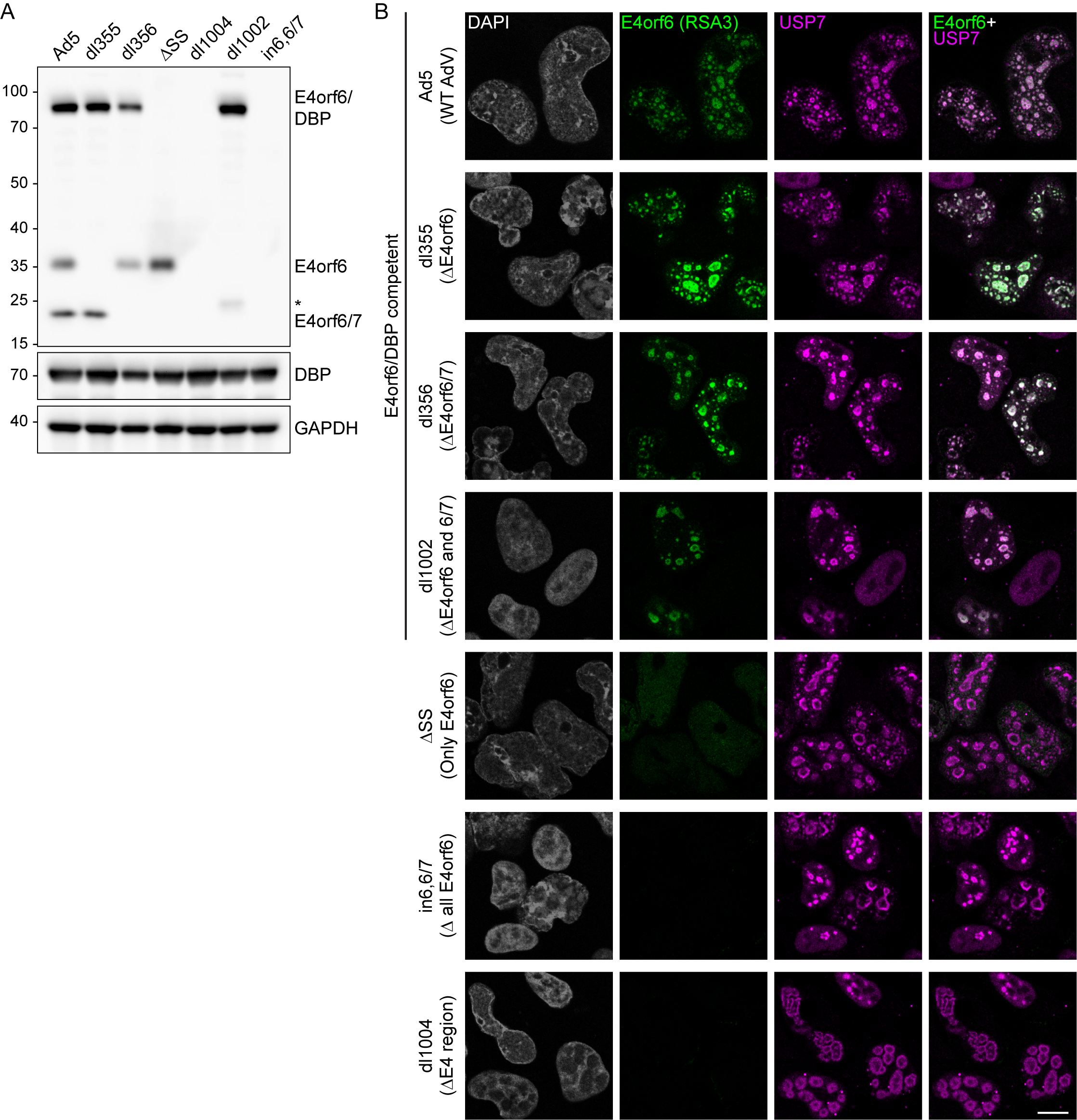

Supplement: S3 Fig — (A) A549 cells were infected with the indicated mutant adenoviruses for 24 hours and proteins were resolved by immunoblot. RSA3 antibody shows various banding patterns for E4orf6/DBP, E4orf6, and E4orf6/7. * denotes E4orf6 frameshift product that is produced downstream of the common E4orf6 splice donor in dl1002. (B) A549 cells were infected with the viruses from A for 24 hours before immunofluorescence was performed. Cells were stained with antibodies against E4orf6 N terminal domain (RSA3, green) or cellular USP7 as a marker of viral replication centers (magenta). White scale bar denotes 10 μm. (TIF) [file ppat.1010797.s005.tif]

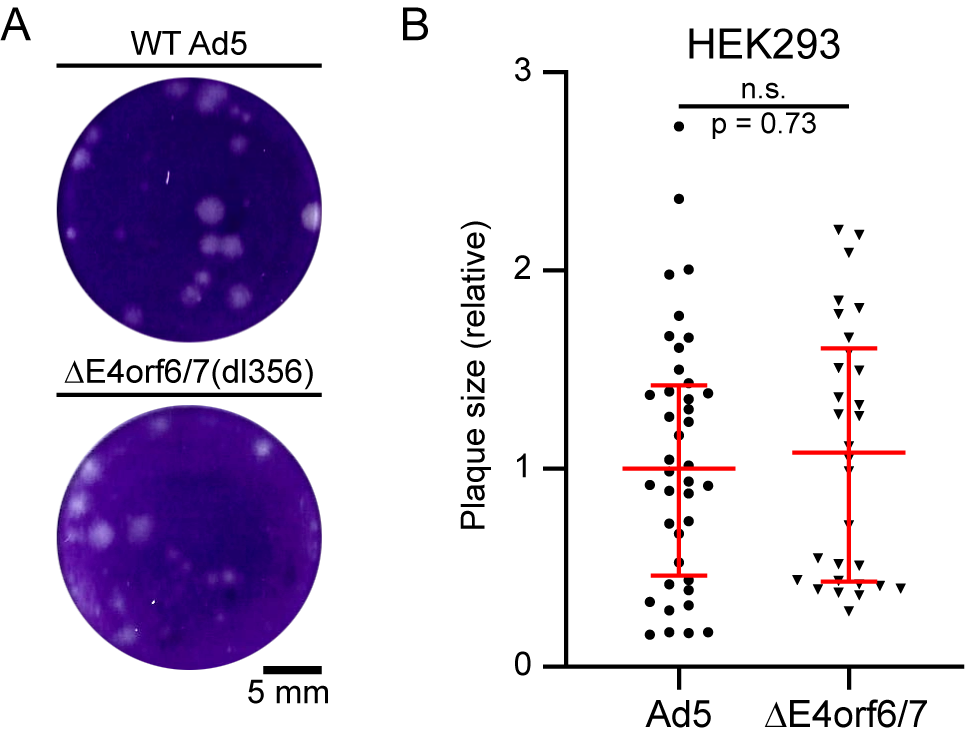

Supplement: S4 Fig — (A) HEK293 cells were infected with limiting dilution of WT Ad5 or ΔE4orf6/7 (dl356) for six days to allow the formation of plaques. Plaque formation was negative stained with crystal violet and imaged. Scale bar shows 5 mm. (B) WT Ad5 or ΔE4orf6/7 plaque size in HEK293 cells was quantified with ImageJ and normalized to the median plaque size in WT infection. Individual plaques are plotted as a single point. Red error bars denote median and interquartile range. Statistical significance was performed using non-parametric Mann-Whitney t-test (n.s., not significant). (TIF) [file ppat.1010797.s006.tif]
