# Supplementary material for: Novel viral splicing events and open reading frames revealed by long-read direct RNA sequencing of adenovirus transcripts
Source: PLoS Pathog. 2022 Sep 12;18(9):e1010797. doi: 10.1371/journal.ppat.1010797 (PMC9499273; doi:10.1371/journal.ppat.1010797)
Supplement: Attachment — Submitted filename: Ad5 PLoS Path Reviewer Comments.pdf [file ppat.1010797.s007.pdf]

Dear Professor Weitzman,

Thank you very much for submitting your manuscript "Novel viral splicing events and open reading frames revealed by long-read direct RNA sequencing of adenovirus transcripts" for consideration at PLOS Pathogens. As with all papers reviewed by the journal, your manuscript was reviewed by members of the editorial board and by several independent reviewers. In light of the reviews (below this email), we would like to invite the resubmission of a significantly-revised version that takes into account the reviewers' comments.

All three reviewers were enthusiastic about the data presented, and highlighted the novelty in the study. However, reviewers also agreed that additional experiments should be done, including comparing the phenotypes observed with the E4orf6/DBP mutant virus with an E4orf6/7 mutant virus and correlating the Rsa3/M45 immunofluorescent staining pattern with the presence of the E4orf6-related fusion proteins in order to more strongly support the contention that E4orf6 is not localized to viral replication centers. Incorporating these additional experiments would strengthen the manuscript and further support conclusions made by the authors. In addition, it is important to address all of the reviewers concerns in the revision and in the response to reviewers.

We cannot make any decision about publication until we have seen the revised manuscript and your response to the reviewers' comments. Your revised manuscript is also likely to be sent to reviewers for further evaluation.

When you are ready to resubmit, please upload the following:

[1] A letter containing a detailed list of your responses to the review comments and a description of the changes you have made in the manuscript. Please note while forming your response, if your article is accepted, you may have the opportunity to make the peer review history publicly available. The record will include editor decision letters (with reviews) and your responses to reviewer comments. If eligible, we will contact you to opt in or out.

[2] Two versions of the revised manuscript: one with either highlights or tracked changes denoting where the text has been changed; the other a clean version (uploaded as the manuscript file).

Important additional instructions are given below your reviewer comments.

Please prepare and submit your revised manuscript within 60 days. If you anticipate any delay, please let us know the expected resubmission date by replying to this email. Please note that revised manuscripts received after the 60-day due date may require evaluation and peer review similar to newly submitted manuscripts.

Thank you again for your submission. We hope that our editorial process has been constructive so far, and we welcome your feedback at any time. Please don't hesitate to contact us if you have any questions or comments.

Sincerely,

Donna Neumann, PhD
Associate Editor

PLOS Pathogens

Karl Münger
Section Editor
PLOS Pathogens

Kasturi Haldar
Editor-in-Chief
PLOS Pathogens

Michael Malim
Editor-in-Chief
PLOS Pathogens

All three reviewers were enthusiastic about the data presented, and highlighted the novelty in the study. However, the reviewers also agreed that additional experiments should be done, including comparing the phenotypes observed with the E4orf6/DBP mutant virus with an E4orf6/7 mutant virus and correlating the Rsa3/M45 immunofluorescent staining pattern with the presence of the E4orf6-related fusion proteins in order to more strongly support the contention that E4orf6 is not localized to viral replication centers.

We would like to thank the editors for compiling these reviews. We have addressed all the comments and believe that our manuscript is substantially improved by the process.

Reviewer's Responses to Questions

Part I - Summary
Please use this section to discuss strengths/weaknesses of study, novelty/significance, general execution and scholarship.

Reviewer #1: Manuscript by Price et al is about identification of novel HAdV5 transcripts by using advanced long-read RNA sequencing approach. The study identifies previously missed SNPs in the HAdV5 genome, re-defines TSS & CPAS, and most importantly identifies at least 35 novel transcripts (out of which ca. half of them encode detectable proteins). The manuscript focuses on one of the novel transcript encoded protein, E4orf6/DBP, and elegantly characterizes the functions of this protein in HAdV infection.

The manuscript is very-well written, the authors include correct references and the experiments are well-controlled. I would also highlight that the images are aesthetically pleasing to look at. All of this is not very surprising considering the previous high-impact and high-standard manuscripts coming from the Dr. Weitzman's lab. A big respect for that.

I can not really find major weaknesses of the study, as the experiments are elegantly done and proved using different experimental approaches. Moreover, some of the sequencing results

partially overlap with the findings from 2 previous long-read sequencing studies (Donovan-Banfield., 2020 & Westergren-Jakobsson.,2021), hence supporting the reproducibility of the authors sequencing data.

The novelty is clearly in the characterization of one of the new proteins (E4orf6/DBP), something that previous sequencing studies did not even consider. Or in other words, we all have missed this protein, although it was always on our western blot images with an anti-E4orf6 antibody! Moreover the authors also make a fundamental finding regarding the E4orf6 subcellular localization, which the adenovirus community has been fooled for decades due to detection of the E4orf6/DBP protein. Additional mapping of the TSS and CPAS will help the adenovirus community to better characterize the synthesis and co-/post-transcriptional processing of the viral transcripts, something that is clearly needed to get a better picture of the HAdV life-cycle. And finally, identification of novel viral transcripts/proteins, which will take now some time to find their function.

Summa summarum: as such the manuscript will not have only a very high impact among the adenovirologists but also among other DNA virus researchers studying the complexity of viral transcriptomes.

We would like to thank the reviewer for their incredibly kind review and summary of our work. We have included the asked for minor changes in our revised manuscript as detailed below.

Reviewer #2: The authors utilize Illumina short-read and nanopore long-read direct RNA sequencing approaches in Adenovirus type 5 (HAdV-C5)-infected A549 cells to identify all viral transcripts including transcription start sites, RNA cleavage, and RNA polyadenylation sites. Several published reports (refs 54 and 55) previously mapped HAdV-C2/5 transcripts during time courses of infection, but the current study identified a number of new transcripts not previously reported. Here, the authors identified 35 new HAdV-C5 viral transcripts including 14 with new splice junctions, 6 novel ORF mRNAs, and 15 mRNAs with splicing patterns that could generate new fusion proteins of annotated ORFs. The authors pursue one such novel fusion ORF that fuses the N-terminal region of E4orf6 with the E2-encoded DNA binding protein (DBP) - termed E4orf6/DBP. They confirm the expression of E4orf6/DBP in infected cells and generate a mutant virus that eliminates its expression. The E4orf6/DBP mutant virus replicates normally and expresses viral proteins equivalently to HAdV-C5 wild-type virus. The authors identify two phenotypes associated with loss of E4orf6/DBP expression: viral nuclear replication centers have an unusual morphology and the virus exhibits a small plaque phenotype. Overall, these studies are convincing and technically at the cutting edge. With this said, the results are highly specific to Adenovirus and thus directed toward a limited readership. Also, the basis of the altered replication center morphology and small plaque phenotype with the E4orf6/DBP viral mutant is unknown.

We would like to thank the reviewer for their comments on our study. We have included new data characterizing the effects of an E4orf6/7 deleted virus in our revised manuscript as detailed below. Although focused on adenovirus, we think that this overall approach of combining short-read and long-read sequencing with proteomics over a time course of infection will be applicable to other viral systems.

Reviewer #3: Long-read (nanopore) and short-read (Illumina) sequencing reveals three dozen novel viral transcripts from human adenovirus type 5. Of note, a novel evolutionarily conserved protein containing the amino-terminus of E4orf6 fused to the E2A DNA-binding protein was

identified. Expression of this conserved fusion protein was eliminated by changing the E4orf6/7 splice-donor site (also eliminating the non-essential E4orf6/7 fusion protein.) The absence of this novel fusion protein was found to have no measurable consequences on viral gene expression or genome replication but may have affected the manner or rate of cell death or virus release.

This work addresses a critical gap in our knowledge. Specifically, the annotation of the type 5 reference strain is incomplete and lacks transcription start sites, RNA cleavage and polyadenylation sites and the associated 5' and 3' non-translated sequences. This work also provides new information on novel adenovirus products that encourage a re-evaluation of our previous understanding of the localization and distribution of key viral proteins during a viral infection.

Recently published work from Mathews (2020) and Akusjarvi (2021) have described the complexity of transcription from adenovirus type 2 and type 5 using long-read methods. This work provides similar information on the transcriptional profile of type 5 adenovirus but includes a comprehensive proteomic analysis and integrated short-read analysis to define transcription start sites as well as polyadenylation and cleavage sites. An unappreciated fusion product was verified in this study at the level of RNA sequencing and protein analysis. The contribution (or lack thereof) of this fusion protein to viral replication was studied. Although this E4orf6/E2A-DPB fusion product appears to have no effect on viral replication, it does appear to contribute to a larger plaque phenotype, consistent with either promoting cell death or virus release.

The quality and the breadth of the data presented here as well as the comprehensive and cogent review of the pertinent literature is a significant strength of this report. Another strength of this work that differentiates this from previously published catalogs of transcriptional changes during adenovirus infection is the coordinated analysis of RNA and protein. The singular limitation I can identify is the overly enthusiastic interpretation that the novel findings here undermine previous held views on adenovirus protein localization with implications for the function of those proteins.

We would like to thank the reviewer for their comments on our study. We have included new data characterizing the localization of various E4orf6 isoforms using additional mutant viruses in our revised manuscript as detailed below.

Part II – Major Issues: Key Experiments Required for Acceptance
Please use this section to detail the key new experiments or modifications of existing experiments that should be absolutely required to validate study conclusions.

Generally, there should be no more than 3 such required experiments or major modifications for a "Major Revision" recommendation. If more than 3 experiments are necessary to validate the study conclusions, then you are encouraged to recommend "Reject".

Reviewer #1: I can not really find any major issues with the paper. Experimentally it is very well conducted study with proper controls and repetitions.

We thank the reviewer for their generous assessment of our work.

Reviewer #2: The E4orf6/DBP mutant virus also does not express the E4orf6/7 protein. The authors mention that an E4orf6/7 mutant virus replicates normally and does not have any obvious phenotype, but does this include an examination of viral replication center morphology and/or plaque size under conditions used in these studies? It seems entirely possible that the phenotypes observed with the E4orf6/DBP mutant virus may partly be due to the loss of E4orf6/7. An E4orf6/7 mutant virus should be examined for comparison.

We thank the reviewer for their constructive comment. We have since acquired the *dl*356 mutant strain of Ad5 which contains a 2bp deletion in E4orf6/7 that results in a non-functional frameshift product while leaving upstream E4orf6 and the novel E4orf6/DBP products intact. In new supplemental data (Figure S3) we show that *dl*356 produces normal viral replication centers when compared to WT Ad5. We have also added Supplemental Figure 4 which shows that the plaque's generated by *dl*356 are not significantly different from WT Ad5 plaques. We believe that these data answer the reviewers concern and substantially improve the manuscript.

Reviewer #3: It is possible that staining at replication centers is dominated by the E4orf6/E2A-DBP fusion protein. If this is so, the viral replication centers in cells infected with E4orf6-deletion virus (dl355) studied by immunoblot in Fig. 6 should show prominent Rsa3/M45 staining patterns at the viral replication centers similar to that seen in cells infected with the wild-type virus. If this is so, this would strongly support the notion that the bulk of that staining pattern is due to the novel fusion protein. I suggest that this experiment be performed along with careful attention to correlating the Rsa3/M45 immunofluorescent staining pattern with the presence of the E4orf6-related fusion proteins in order to more strongly support the contention that E4orf6 is not localized to viral replication centers. The statement that previous reports suggesting localization of E4orf6 to the viral replication was incorrect may be a bit overstated without additional evidence.

We thank the reviewer for their constructive comments. To address the subcellular localization of exactly which E4orf6 isoforms are present within the nucleus or viral replication centers using the N-terminal targeted RSA3 antibody, we have performed additional immunofluorescence using multiple E4 mutant adenoviruses. To address the reviewer's direct point, we have included infection with ΔE4orf6 virus (*dl*355) in main Figure 6 where we compare RSA3 staining of WT Ad5 infection and ΔSS infection. In the total absence of E4orf6 (*dl*355) RSA3 still detects prominent staining at VRCs. In contrast, the ΔSS virus which only retains E4orf6 expression shows broadly nuclear RSA3 staining, with no particular enrichment at VRCs.

In addition, we have included new data in the form of Supplemental Figure 3 which includes RSA3 immunoblot and immunofluorescence using additional Ad5 E4 mutants that lack various E4orf6 isoforms. Infection with *dl*356 (which lacks E4orf6/7) and *dl*1002 (which lacks both E4orf6 and 6/7, but retains E4orf6/DBP due to the mutation being downstream of the splice donor) both retain prominent staining of RSA3 at VRCs. In contrast, E4in6,6/7 loses all RSA3 staining due to a frame-shifting insertion in the upstream E4orf6 exon common to all isoforms. Similarly, *dl*1004 loses all RSA3 staining due to a large deletion removing the majority of the E4 genes including the E4orf6 splice donor. We believe these new data strongly support our hypothesis that RSA3 staining at VRCs is primarily due to the novel E4orf6/DBP isoform. We have adjusted our language in the manuscript so that our findings are not overstated.

The statement in lines 376-377, "Now that we know a major fraction of the E4orf6 signal detected by Rsa3 or M45 during a viral infection is actually E4orf6/DBP…" is somewhat misleading when used to re-interpret previous published results on the localization of E4orf6.

Specifically, this report (Fig. 4E) shows that that this novel fusion form is not present at 16 h postinfection and appears to be lower in abundance compared to the E4orf6 form at 24 h postinfection. The implication that “a major fraction of the E4orf6 signal” is due to the fusion protein needs to be qualified accordingly by noting that this can be true only at significantly late times of infection. Previously conclusions from reports describing the localization of E4orf6 at or before 16 h postinfection are not negated by this report.

The reviewer makes an excellent point that since E4orf6/DBP appears to be a late gene, we cannot make claims for early times post-infection when E4orf6 is expressed in the absence of the chimera. While we did not perform any additional time-course IFA, we have explicitly highlighted uninfected, early-infected, and late-infected cells in our 24 hour post-infection imaging snapshots on the basis of VRC morphology. In Figure 6, uninfected cells (open arrow) show no RSA3 staining as expected. Early-infected cells (closed arrow) show clear, albeit small, VRCs and would be expected to be expressing other early genes such as E4orf6. In these cells we see no staining of RSA3 at VRCs in the presence (WT Ad5) or absence (dl355) of E4orf6, yet sometimes we observed very faint nucleoplasm staining consistent with our assertion for E4orf6’s primary localization.

Part III – Minor Issues: Editorial and Data Presentation Modifications
Please use this section for editorial suggestions as well as relatively minor modifications of existing data that would enhance clarity.

Reviewer #1: I have the following minor comments:

L218-219: I strongly suggest to make Supplementary table where the authors will point out the exact nucleotides they have identified as the TSS and CPAS. Something that was done in the paper by Donovan-Banfield., 2020 (the table is in the supplementary part in their paper). The present figure 2 is nice to look at but does not tell the exact sites/nucleotides as it would be important to compare it with the study by Donovan-Banfield., 2020. Further, the exact TSS & CPAS will help to study the details of HAdV RNA transcription and processing.

Extra: In the paper by Donovan-Banfiled., the authors indicated that TSS identified by the Nanopore sequencing was ~8–15 nucleotides downstream of the previously established locations. That was regarded as a Nanopore sequencing specific phenomena/artefact. So, it would be very informative to know if the Price et al., also saw the similar phenomena? Is that taken into consideration when you were defining the TSS? Again, the supplementary table with exact nt. for TSS and CPAS would help a lot here.

We have now included a supplementary table (Table S1) that includes exact nucleotides for TSS and CPAS. We also see nanopore dRNA reads terminating downstream of the actual 5’ TSS, which is a known artifact based on the nanopore pore size and inability of the motor to pump the last few nucleotides through. In Table S1 we have included both the exact TSS peak called by our algorithm, as well as the manually adjusted peak we used in our annotation based on integrating short-read Illumina data. Our average nucleotide discrepancy was 10.5 nt, in line with previously published reports.

L300: to be honest, I can not see the "increase dramatically" effect for the E4-DPB and E4orf6/DBP due to low resolution of the graphs. I would suggest to include numbers for fold increase (12hpi vs 24hpi) after each transcript (smth like: 19K/IX(4x), E4-DPB(Xx), E4orf6/DBP(Xx)) to get a better idea about the increase. And avoid word "dramatically"....

Fold changes have been added to the text and the word dramatically was removed.

L401: I suggest to point out that the loss of E4orf6 had a major effect at 24hpi, and partial effect at 48hpi based on the provided image. To honest, I do not see major effect of E4orf6 deletion on hexon and fiber at 48hpi.

We have changed the text to reflect these more accurate descriptions.

L403: it is overstatement, consider using "partial loss of degradation" as based on the provided image Rad50 level is still less in E4orf6 lacking cells when compared to mock cells.

Done.

L487: not entirely true considering 2 previous studies by Donovan-Banfield., 2020 & Westergren-Jakobsson.,2021. Consider rephrasing the sentence.

We have added additional discussion of both prior adenovirus nanopore transcriptome papers to the Introduction and Discussion of the manuscript.

L502-510: The study Westergren-Jakobsson.,2021 also identified SNPs in the HAdV2 with Nanopore sequencing. The authors should compare, and discuss, the SNPs found in their study to the ones found by Westergren-Jakobsson.,2021. Are there any overlaps?

While HadV5 and HadV2 are very similar at the protein level, their respective genomes carry a large number of variants at the nucleotide level, making such an interpretation of any given SNP difficult. However, none of the SNPs we discovered in HAdV5 were identical, or even from the same region, as those previously reported by Westergren-Jakobssen for HAdV2.

Discussion in general: the authors are modest in describing the previous studies by Donovan-Banfield., 2020 & Westergren-Jakobsson.,2021. (L516). It would be more informative if the authors can discuss if any of their novel 35 transcripts were also identified in these 2 previous studies. Or at least comment if the E4orf6/DBP transcript was detected in HAdV2/IMR90 (Westergren-Jakobsson.,2021.) and HAdV5/MRC-5 (Donovan-Banfield., 2020) virus/cell systems. That may give a hint about the universal nature of the E4orf6/DBP transcript in different cell systems.

We have included a new supplementary table (Table S2) where we highlight whether our 35 high-confidence transcripts were also present in either the Donovan-Banfield or Westergren-Jakobssen studies. Excitingly, 33/35 transcripts were shared between both Ad5 studies, and 28/35 being shared between Ad5 and Ad2. In addition, an extra 2 transcripts might have been shared between Ad5 and Ad2, but the Westergren-Jakobssen supplemental data did not include coordinates for intra-TPL isoforms that splice downstream to canonical L1-L5 transcripts. We now discuss these comparisons in the results and discussion section.

Reviewer #2: The authors conclude on line 386 that E4orf6/DBP has "different cellular localizations, binding partners, and functions compared to E4orf6." They clearly show overt differences in subcellular localization, but on what basis do they conclude that E4orf6/DBP has different binding partners and functions? They can discriminate binding of E4orf6 with E1B55K protein and Mre11/Rad50 degradation, but this does not mean E4orf6/DBP has novel binding partners and function.

We agree that we have not fully addressed unbiased binding partners or overlapping functions outside of the viral ubiquitin ligase. We have changed our concluding line to read “Thus, E4orf6/DBP has different cellular localizations compared to E4orf6, and does not bind to E1B55K or participate in ubiquitin ligase activities.”

Reviewer #3: Localization of the FLAG-tagged E4orf6 variants is another concern. The results presented in Suppl. Fig 2 were argued to support the notion that E4orf6 is diffusely localized in the nucleus and does not strongly association with viral replication centers. How long after infection were these result analyzed and how well does the retroviral construct recapitulate the time course of expression of the various E4 and E2A-DBP forms?

The figure legend for S2 has been updated to show that IFA was performed 24 hpi in the retroviral transduced cells. Temporal expression of the flag-tagged E4orf6/DBP can be seen in the RSA3 blot of Figure 6C and directly compared to the endogenous protein due to the molecular weight shift of the larger 2x-flag product. The retroviral transgene might be expressed slightly more at early times post infection (16 hpi) but appears to match the endogenous expression of E4orf6/DBP at 24 and 48 hpi.

Another concern is that anecdotal evidence has suggested that N-terminal modifications of E4orf6 create a non-functional protein. Is the FLAG-tagged E4orf6 protein studied here known to be functional with respect to viral replication (and not simply reconstituting the ubiquitin protein ligase?)

We are unaware of the anecdotal evidence about N-terminal modifications creating non-functional E4orf6. Nevertheless, in Figure 5B and 5D we were able to show that flag-E4orf6 is able to reconstitute ubiquitin ligase activity and binding to E1B55K as mentioned by the reviewer. While we did not test the ability of flag-E4orf6 to rescue a ΔE4orf6 infection, flag-E4orf6/DBP was sufficient to rescue both of the phenotypes we have associated with the novel chimeric protein.

How does the failure of E1B-55K to localize to viral replication centers in the absence of E4orf6 reconcile with the absence of E4orf6 at the viral replication centers?

We cannot speculate about the localization of E1B55K during infection with our various E4 mutant viruses. However, as can be noted in the new Supplemental Figure 3, viral replication centers in the absence of E4orf6 are different from both wildtype infection and the novel phenotype we associate with loss of E4orf6/DBP. It could be possible that E4orf6 is involved in regulating subnuclear morphology without being directly associated with VRCs.

Any speculation on why internal L4 promoter identified by Leppard not identified in A549 cells?

Our cutoffs for calling a TSS or CPAS involved the site being reliably detected by nanopore sequencing at both 12 and 24 hpi. The L4 promoter was robustly detected at 12 hpi, but lost to

the noise of splice-site associated “false TSS’s” at 24 hpi. As such, we have updated Figure 2 to include a bracketed TSS annotation for the L4 promoter and comment on this in the text and methods.
